# Withholding and withdrawing life-support in adults in emergency care: joint position paper from the French Intensive Care Society and French Society of Emergency Medicine

**DOI:** 10.1186/s13613-019-0579-7

**Published:** 2019-09-23

**Authors:** Jean Reignier, Anne-Laure Feral-Pierssens, Thierry Boulain, Françoise Carpentier, Pierrick Le Borgne, Denis Del Nista, Gilles Potel, Sandrine Dray, Delphine Hugenschmitt, Alexandra Laurent, Agnès Ricard-Hibon, Thierry Vanderlinden, Tahar Chouihed, For the French Society of Emergency Medicine (Société Française de Médecine d’Urgence, SFMU) and French Intensive Care Society (Société de Réanimation de Langue Française, SRLF)

**Affiliations:** 1 Service de Médecine Intensive Réanimation, Centre Hospitalier Universitaire Hotel-Dieu, 30 Bd. Jean Monnet, 44093 Nantes Cedex 1, France; 2 Université de Nantes, Nantes, France; 3 Assistance Publique Hôpitaux de Paris, Service des Urgences, Hôpital Européen Georges Pompidou Paris, Paris, France; 4 Service de Réanimation Médicale Polyvalente, Centre Hospitalier Régional Orléans, Orléans, France; 5 Pôle Urgences Médecine Aigüe, Hôpital Universitaire des Alpes, Grenoble, France; 6 Université de Grenoble, Grenoble, France; 7 Service d’Accueil des Urgences, Hôpital de Hautepierre, CHRU Strasbourg, Strasbourg, France; 8 Service des Urgences, CH de Rochefort, Rochefort, France; 9 Service des Urgences, CHU de Nantes, Nantes, France; 10 Service de Réanimation Médicale, Hôpital Nord, CHU de Marseille, Marseille, France; 11 SAMU-SMUR de Lyon, Hospices Civils de Lyon, Lyon, France; 12 Laboratoire Psy-DREPI, Université de Bourgogne Franche-Comté, EA7458, Dijon, France; 13 SAMU-SMUR 95- Service des Urgences, Centre Hospitalier René Dubos, Pontoise, France; 14 Service de Réanimation Polyvalente, Groupe Hospitalier Institut Catholique de Lille/Faculté Libre de Médecine/Université Lille Nord de France, Lille, France; 15 SAMU-SMUR-Service d’Urgences, Hôpital Central, CHRU Nancy, Vandoeuvre les Nancy, France; 16 INSERM U1116, Université de Lorraine, Vandoeuvre les Nancy, France

## Abstract

For many patients, notably among elderly nursing home residents, no plans about end-of-life decisions and palliative care are made. Consequently, when these patients experience life-threatening events, decisions to withhold or withdraw life-support raise major challenges for emergency healthcare professionals. Emergency department premises are not designed for providing the psychological and technical components of end-of-life care. The continuous inflow of large numbers of patients leaves little time for detailed assessments, and emergency department staff often lack training in end-of-life issues. For prehospital medical teams (in France, the physician-staffed mobile emergency and intensive care units known as SMURs), implementing treatment withholding and withdrawal decisions that may have been made before the acute event is not the main focus. The challenge lies in circumventing the apparent contradiction between the need to make immediate decisions and the requirement to set up a complex treatment project that may lead to treatment withholding and/or withdrawal. Laws and recommendations are of little assistance for making treatment withholding and withdrawal decisions in the emergency setting. The French Intensive Care Society (*Société de Réanimation de Langue Française*, SRLF) and French Society of Emergency Medicine (*Société Française de Médecine d’Urgence*, SFMU) tasked a panel of emergency physicians and intensivists with developing a document to serve both as a position paper on life-support withholding and withdrawal in the emergency setting and as a guide for professionals providing emergency care. The task force based its work on the available legislation and recommendations and on a review of published studies.

## Introduction

For several decades, the public, medical community, scientific societies, and law makers have been emphasizing the right of patients to decide which treatments they receive and to die with dignity. Legislation and recommendations in France reject futile therapeutic interventions and require that physicians document the wishes of the patient (via advance directives if the patient is unconscious), follow a collegial decision-making procedure when appropriate, and provide palliative care including, if needed, deep sedation at the end of life [1–7].

Laws and recommendations are of little assistance, however, for making treatment withholding and withdrawal decisions in the emergency setting. Of the 600,000 people who die each year in France, nearly 60% die in healthcare institutions. In 16% of these cases, the patient dies within 24 h of admission, often in the emergency department. Among patients admitted to emergency departments, 0.1–0.2% die there, accounting for 5–7% of all in-hospital deaths. In elderly institutionalized patients, about one quarter of deaths occur in the hospital [8–10].

The mismatch between regulatory requirements and everyday reality is glaring. Most patients, even among people with severe chronic diseases, neither communicate their wishes in detail to their family or physician nor establish advance directives. Thus, for many patients, notably among elderly nursing home residents, no plans about end-of-life decisions and palliative care are made. Consequently, when these patients experience life-threatening events, decisions to withhold or withdraw life-support raise major challenges for emergency healthcare professionals. The patient’s main doctor—if one has been designated—is rarely available for consultation on an emergency basis. Furthermore, setting up a collegial decision-making procedure that complies with legislative requirements faces extraordinary practical obstacles in the emergency setting (including during prehospital care).

Healthcare teams often feel powerless in this situation. Emergency department premises are not designed for providing the psychological and technical components of end-of-life care. The continuous inflow of large numbers of patients leaves little time for detailed assessments, and emergency department staff often lack training in end-of-life issues. For prehospital medical teams (in France, the physician-staffed mobile emergency and intensive care units known as SMURs), implementing treatment withholding and withdrawal decisions that may have been made before the acute event is not the main focus. The challenge lies in circumventing the apparent contradiction between the need to make immediate decisions and the requirement to set up a complex treatment project that may lead to treatment withholding and/or withdrawal. Neither the current legislation nor the latest recommendations provide clear answers to this conundrum. Consequently, the French Intensive Care Society (*Société de Réanimation de Langue Française*, SRLF) and French Society of Emergency Medicine (*Société Française de Médecine d’Urgence*, SFMU) tasked a panel of emergency physicians and intensivists with developing a document to serve both as a position paper on life-support withholding and withdrawal in the emergency setting and as a guide for professionals providing emergency care [11]. The task force based its work on the available legislation and recommendations and on a review of published studies, a selection of which is given at the end of this article [12–43].

### Question 1: what are the key considerations when making withholding/withdrawal decisions in the emergency setting?

- First, information must be sought about the patient’s wishes, either directly if the patient is competent and able to communicate or indirectly via the patient’s advance directives or, if none exist, by interviewing the patient’s healthcare proxy or family [7].
- When no information is available on the patient’s wishes, treatment limitation decisions must not be based on age alone.
- Every effort must be made to obtain the patient’s complete medical file, together with information about previous self-sufficiency, cognitive function, and quality of life. In patients with potentially fatal advanced chronic diseases, factors relevant to decisions about the appropriate level of treatment include both a history of previous impairments (e.g., profound debilitation, cachexia, dependency, or incapacitating cognitive disorders) and the availability of curative treatments.
- Expected treatment benefits should be weighed against treatment-related burdens and the potential adverse impact of treatments and/or ICU management.
- Routine ICU admission does not contribute to improve the survival or quality of life of very elderly patients [24]. Withholding/withdrawal decisions are not consistently followed by death of the patient. Self-sufficiency and frailty are key factors in treatment limitation decisions. The self-sufficiency classification system used in France to distinguish six levels of assistance needed by elderly people (GIR) can help in the decision-making process. Patients in group 1,^1^ who have the highest level of dependency, are generally eligible for comfort care only, without life-supporting interventions. Unless the cause of the life-threatening event can be simply and rapidly reversed, patients with dementia who are classified in group 2^2^ should be managed in the same way as group 1 patients.
- In patients with acute events that severely threaten survival and function and may require unusual treatments (e.g., decompressive hemicraniectomy for malignant cerebral infarction), intensive management is warranted to provide time to collect the necessary information. Thus, the level of care must be discussed on a case-by-case basis with the patient, or the family if the patient is incompetent, and with the intensivist [34].

### Question 2: what are the key considerations when developing a treatment plan in the emergency setting?

- Each emergency department must have a written protocol that is available at all times and describes the principles and modalities of the decision-making process and the management of dying patients admitted to the emergency department [6, 7, 29].
- If the patient is able to make decisions and communicate wishes, the staff must allow sufficient time to listen to those wishes and decisions and to obtain relevant information, notably on the available treatment options.
- If the patient is unable to communicate, information should be sought from advance directives or, if none are available, from the family [13]. When deemed appropriate for the current situation, advance directives must dictate all decisions about investigations, procedures, and treatments. In the emergency setting, however, healthcare professionals may disregard advance directives that they deem inappropriate for the current situation or that the family is unwilling to have applied. The physician may then decide to provide intensive care until the relevance of the advance directives can be confidently assessed.
- In every case, a collegial discussion among all physicians and other healthcare professionals present must be held [23].
- Efforts to achieve collegiality should not result in the provision of futile care.
- All interviews, discussions, and decisions must be recorded in the patient’s medical file. Recording only the decisions is not sufficient; instead, the process by which the decisions were built must be described, as well as the modalities for implementing the decisions.
- Caution is in order in patients with previous withholding and/or withdrawal decisions. These should always be reevaluated and can be implemented only if deemed appropriate for the new situation and consonant with the patient’s wishes.

### Question 3: provisional intensive care: what is it and when is it appropriate?

- Provisional intensive care consists in providing life-support to protect the patient from loss of chance when time is needed to resolve uncertainties about a life-threatening situation [6, 43].
- Provisional intensive care may be supplied for several hours, after which a reappraisal may be performed (e.g., once the diagnosis has been confirmed, the previous health status clarified, and the response to treatment assessed; or until the family arrives and can be prepared to receive distressing news).
- Provisional intensive care can be initiated at the prehospital phase and does not require routine ICU admission but can, instead, be continued in the resuscitation area of the emergency department [20].
- If uncertainties persist after several hours, further investigations and active treatments should be provided either in the ICU or in an intensive monitoring unit, depending on the level of care required.

### Question 4: how can organ donation be organized in the emergency setting [42]?

- A patient with severe brain damage that is not amenable to treatment and that may result in brain death should be viewed as a potential donor regardless of age.
- The family members should be informed that brain death is a possible outcome, so that they can prepare for the imminent death of their loved one, provide any information they may have about his or her wishes, and consider medical transport of the patient to a center with expertise in organ harvesting.

### Question 5: what are the requirements for establishing collegiality in the emergency setting [1–4, 12, 28, 37]?

- During the office hours, the requirements set down by law must be met. The patient and family members must be involved, together with the physician and other healthcare staff present at the time and with an external consultant having relevant expertise, who may be the general practitioner, a referral specialist, or an intensivist.
- Outside office hours, limited collegiality is acceptable, with the involvement of the patient and family members, the physician and other healthcare staff present at the time and, whenever possible, a physician whose advice seems highly relevant to the situation at hand and who may be consulted either in person or by telephone.

### Question 6: how can high-quality communication with family members be reconciled with the specific constraints imposed by the emergency setting?

- When considering treatment withholding and/or withdrawal decisions, holding a meaningful discussion with the family members is challenging for the physician in charge, who is often insufficiently equipped for this task and concerned about generating intense emotional reactions. This situation is highly asymmetrical: the physician is performing a professional task based on a sound technical understanding of the situation, whereas the family member is in a state of uncertainty and is going through an extraordinary experience that may change the course of her or his life. Although nothing can soften the nature of the information that must be imparted, the content and shape of the interview should be anticipated [13, 33].
- The following rules must be applied [43]:
  - The interview should take place in a welcoming, quiet, and private room in which all the individuals present can be seated.
  - The nurse and nursing assistant in charge of the patient should be present, together with the psychologist of the department if possible.
  - Interruptions from outside sources should be avoided. If any are expected, this should be disclosed to the family before starting the interview.
  - The interview must be conducted as follows:
    - The staff must show kindness, empathy, availability, and a willingness to listen.
    - Each healthcare staff member present should be introduced to the family.
    - The family should be informed that the final decision is the sole responsibility of the healthcare team (“To make the best decision and to act in the best interests of your loved one, we need to understand your loved one’s wishes regarding the current situation, and you can help us by telling us what those wishes are.”).
    - Use both verbal and nonverbal communication methods appropriate for the situation.
    - Start by asking questions about what the family already knows then gradually add further information, while allowing enough time for the family to integrate the new elements (regulate the speech rate, pause when appropriate, reformulate, and ask questions to assess comprehension), understand and respect any reactions, and help the family members ask or word their questions and vent their emotions.
    - Avoid technical terms.
    - Bring the interview to a close and offer support to the family members.
- Conducting the interview over the telephone can result in misunderstandings and conflicts and is, therefore, inadvisable. If a telephone interview is unavoidable, the same principles as for the face-to-face interview must be applied. Before starting the interview, the identity of the interlocutor and his or her relationship to the patient must be determined, as well as whether the interlocutor is capable of having this important conversation.
- During the prehospital phase, every effort should be made to meet these conditions as closely as possible.

### Question 7: how should life-support withdrawal be organized in the emergency department?

- Treatment withdrawal modalities should not be affected in any way by the emergency setting.
- In a patient initially managed with invasive life-sustaining interventions such as endotracheal and/or vasoactive agents, these can be withdrawn secondarily in the emergency department.
- Given the usually short time from life-support withdrawal and/or deep sedation initiation to death, holistic care should be provided by the emergency department team, at the most appropriate location (e.g., resuscitation area or short-stay unit), to ensure that continuous care is provided to the patient and family.
- Patients who do not die rapidly after life-support withdrawal should be admitted to a ward, preferentially in a department where the patient has already received care or in the palliative care unit. The care plan should be described in detail in the patient’s medical file and communicated unambiguously to all teams involved in providing care to the patient.

### Question 8: how should deep sedation be initiated in the emergency setting?

- Deep sedation should be initiated as soon as possible in dying patients who are comatose or who remain in pain or distress despite optimal treatment [12].
- Deep sedation should be achieved by combining a hypnotic agent with an opioid analgesic [6, 43].
- The effectiveness of the sedation and analgesia should be assessed regularly and the dosages adjusted to the predefined targets.
- The deep sedation modalities and effects should be recorded in detail in the patient’s medical file.
- The physician should make sure that the family members understand the methods and goals of deep sedation and that they receive optimal support.

### Question 9: what is the role for palliative care in the emergency setting?

- End-of-life care, even in the emergency setting, must comply with clinical practice guidelines for palliative care [30].
- The emergency department environment is not conducive to the optimal management of end-of-life situations. The least unsatisfactory place for temporarily providing more appropriate conditions is the resuscitation area or a short-stay unit.
- All interventions likely to improve patient comfort must be provided including appropriate nursing care, treatments to control pain and dyspnea, and respiratory secretion clearance.
- All interventions that fail to enhance patient comfort or may cause patient discomfort should be stopped. Examples include vital sign monitoring, laboratory tests, and capillary blood glucose monitoring.
- Supplemental oxygen therapy is generally useless and should be provided only if it improves patient comfort.
- Nutrition and fluids should be withheld unless requested by the patient. Few patients report being thirsty; limiting the fluid intake, in combination with antisecretory agents if appropriate, helps to combat secretion build-up.
- Palliative care specialists can be asked for input in the emergency setting and can subsequently contribute to provide feedback and debriefing about challenging experiences.
- Efforts should be made to train physicians and other healthcare professionals in the provision of palliative care in the emergency setting.

### Question 10: can treatment withholding and withdrawal decisions be made at the prehospital phase of patient care?

- Although prehospital care has specific characteristics, the ethical principles of beneficence, non-maleficence, respect for autonomy, and justice should be applied under all circumstances [19].
- The above-described modalities of the decision-making process apply to the prehospital setting.
- Prehospital management by physicians can provide the opportunity to either initiate or discontinue life-supporting treatments when the appropriate conditions are met (availability of complete information and feasibility of palliative care) [19, 20].
- Nevertheless, in challenging situations, provisional intensive care can be given and the patient taken to a hospital to provide enough time to obtain clarity in complex cases.
- Dying patients who receive prehospital care are not necessarily taken to an emergency department or even a hospital [19, 21].
- A physician-staffed mobile emergency unit (SMUR) can provide treatment for a life-threatening situation and subsequently initiate palliative care after discussion with the coordinating physician.
- A physician-staffed mobile emergency unit (SMUR) should not be asked to intervene if optimal palliative care can be rapidly provided on-site, e.g., in a nursing home.

### Question 11: who should be involved in prehospital decisions (in person or over the telephone)? [21]

- The patient and family.
- The coordinating physician at the dispatch center plays a pivotal role.
- The members of the prehospital healthcare team.
- The general practitioner, or the referral specialist if possible.
- The healthcare professionals in a network or residential facility, if any is involved.
- The intensivist or emergency physician who may be called on to manage the patient.

### Question 12: how can palliative care be organized in the prehospital setting? [19–21]

- Careful attention should be directed to communicating with the family members, the professionals providing care on-site, and the general practitioner or referral physician, with the goals of ensuring that they agree with the care plan and of protecting the patient from discomfort, notably by avoiding unnecessary transportation to an emergency department.
- A physician-staffed mobile emergency unit initially called to treat a life-threatening situation may, after determining that the patient is at the end of life, initiate deep sedation [19, 20]. Follow-on care by the available healthcare professionals should be arranged.
- The mobile unit-coordinating physician should be able to send the mobile team to another patient if necessary and should, therefore, be continuously and actively involved in the process. The intervention of the mobile team is then curtailed and further care provided, in a hospital if needed.
- Information on the management of the patient must be detailed in the medical file and communicated to all the physicians involved the patient’s management to ensure continuity of care [21].

## Data Availability

Not applicable.

## Footnotes

1 Patient confined to bed or chair, with severe cognitive dysfunction, requiring continuous care from others; or dying patient.

2 Patient confined to bed or chair, with some residual cognitive function, requiring assistance for most daily activities; or patient with dementia who can move around but must be watched at all times.

## References

[1] Conseil de l’Europe, Assemblée parlementaire. Protection des droits de l’homme et de la dignité des malades incurables et des mourants. Recommandation n°1418. 1999. http://assembly.coe.int/nw/xml/XRef/Xref-XML2HTML-FR.asp?fileid=16722&lang=FR. Dernier accès 20 Apr 2018.

[2] République française. Loi n°2002-303 relative aux droits des malades et à la qualité du système de santé du 4 mars 2002. J Off République Française. 2002. https://www.legifrance.gouv.fr/affichTexte.do?cidTexte=JORFTEXT000000227015&categorieLien=id. Dernier accès 20 Apr 2018.

[3] République française. Loi n° 2005-370 du 22 avril 2005 relative aux droits des malades et à la fin de vie. J Off République Française. 2005. https://www.legifrance.gouv.fr/affichTexte.do?cidTexte=JORFTEXT000000446240&categorieLien=id. Dernier accès 20 Feb 2018.

[4] République française. Loi n° 2016-87 créant de nouveaux droits en faveur des malades et des personnes en fin de vie. J Off République Française. 2016. https://www.legifrance.gouv.fr/affichTexte.do?.

[5] Société Française de Médecine d’Urgence (2003). Ethique et urgences réflexions et recommandations. JEUR.

[6] Société de Réanimation de Langue Française (2010). Limitation et arrêt des traitements en réanimation adulte. Actualisation des recommandations de la SRLF. Réanimation.

[7] Downar J, Delaney JW, Hawryluck L, Kenny L (2016). Guidelines for the withdrawal of life-sustaining measures. Intensive Care Med.

[8] Rothmann C, Evrard D (2005). La mort aux urgences. JEUR.

[9] Lalande F, Veber O. La mort à l’hôpital. Rapport Inspect Gén Affaires Soc. 2009; RM2009-124P: 164p http://www.ladocumentationfrancaise.fr/rapports-publics/104000037/index.shtml. Dernier accès 20 Apr 2018.

[10] Volant S (2014). 693 000 résidents en établissements d’hébergement pour personnes âgées en 2011. Études Résultats.

[11] Feral-Pierssens AL, Boulain TH, Carpentier F (2018). Limitations et arrêts des traitements de suppléance vitale chez l’adulte dans le contexte de l’urgence. Méd Intensive Réanimation.

[12] Adams JA, Bailey DE, Anderson RA, Docherty SL (2011). Nursing roles and strategies in end-of-life decision making in acute care: a systematic review of the literature. Nurs Res Pract.

[13] Azoulay E, Pochard F, Chevret S (2004). Half the family members of intensive care unit patients do not want to share in the decision-making process: a study in 78 French intensive care units. Crit Care Med.

[14] Baile WF, Buckman R, Lenzi R (2000). SPIKES—a six-step protocol for delivering bad news: application to the patient with cancer. Oncologist.

[15] Bioy A (2012). L’aide-mémoire de psychologie médicale et psychologie du soin.

[16] Ciais JF, Pradier C, Ciais C (2007). Impact d’une équipe d’urgence spécialisée sur les hospitalisations non désirées de patients en phase terminale à domicile. Presse Med.

[17] Couilliot M-F, Vassy C, Leboul D (2011). Le temps du mourir et le temps de l’hôpital: prise en charge des patients en fin de vie aux Urgences. Sante Pub.

[18] Curtis JR, Treece PD, Nielsen EL (2008). Integrating palliative and critical care: evaluation of a quality-improvement intervention. Am J Respir Crit Care Med.

[19] Dolveck F, Puybasset L (2010). Questions éthiques liées à la pratique de la médecine d’urgence préhospitalière au SAMU et au SMUR. Enjeux éthiques en réanimation.

[20] Duchateau FX, Beaune S, Ricard-Hibon A (2010). Prehospital noninvasive ventilation can help in management of patients with limitations of life-sustaining treatments. Eur J Emerg Med.

[21] Ferrand E, Marty J, French LATASAMU group (2006). Prehospital withholding and withdrawal of life-sustaining treatments. The French LATASAMU survey. Intensive Care Med.

[22] Ferrand E, Jabre P, Vincent-Genod C (2008). Circumstances of death in hospitalized patients and nurses perception—French multicenter “Mort-à-l’hôpital” survey. Arch Intern Med.

[23] George NR, Kryworuchko J, Hunold KM (2016). Shared decision making to support the provision of palliative and end-of-life care in the emergency department: a consensus statement and research agenda. Acad Emerg Med.

[24] Guidet B, Leblanc G, Simon T (2017). Effect of systematic intensive care unit triage on long-term mortality among critically ill elderly patients in France: a randomized clinical trial. JAMA.

[25] Kentish-Barnes N, Chahraoui K, Laurent A, Bioy A (2015). Communication et accompagnement en situation de fin de vie: le point de vue des familles. Vulnérabilité psychique et clinique de l’extrême en réanimation.

[26] Kentish-Barnes N, Azoulay E (2016). End-of-life care in the ICU: semper ad meliora (always strive for improvement). Intensive Care Med.

[27] Lakin JR, Isaacs E, Sullivan E (2016). Emergency physicians’ experience with advance care planning documentation in the electronic medical record: useful, needed, and elusive. J Palliat Med.

[28] Laurent A, Bonnet M, Capellier G (2017). Emotional impact of end-of-life decisions on professional relationships in the intensive care unit: an obstacle to collegiality?. Crit Care Med.

[29] Le Conte Ph, Guilbaudeau S, Batard E (2005). Mise en place d’une procédure de limitation ou d’arrêt des soins actifs dans un service d’urgence. JEUR.

[30] Le Conte P, Riochet D, Batard E (2010). Death in emergency departments: a multicenter cross-sectional survey with analysis of withholding and withdrawing life support. Intensive Care Med.

[31] McAndrew NS, Leske JS (2015). A balancing act: experiences of nurses and physicians when making end-of-life decisions in intensive care units. Clin Nurs Res.

[32] Picard Y, Leheup BF, Piot É (2016). Limitation et arrêt de traitement: collaboration entre une équipe mobile de soins palliatifs et un service de réanimation. Med Palliat Soins Support Accomp Ethique.

[33] Pochard F, Azoulay E, Chevret S (2001). Symptoms of anxiety and depression in family members of intensive care unit patients: ethical hypothesis regarding decision-making capacity. Crit Care Med.

[34] Quill TE, Holloway RG (2012). Evidence, preferences, recommendations—finding the right balance in patient care. N Engl J Med.

[35] Reignier J, Dumont R, Katsahian S (2008). Patient-related factors and circumstances surrounding decisions to forego life-sustaining treatment, including intensive care unit admission refusal. Crit Care Med.

[36] Reignier J, Cottereau A, Vinatier I. Limitations et arrêts des traitements ou réanimation d’attente? 2017. https://sofia.medicalistes.fr/spip/IMG/pdf/Limitation_et_arrets_des_traitements_ou_reanimation_d_attente.pdf. Accessed 20 Apr 2018.

[37] Sedillot N, Holzapfel L, Jacquet-Francillon T (2008). A five-step protocol for withholding and withdrawing of life support in an emergency department: an observational study. Eur J Emerg Med.

[38] Siddiqui S (2016). A physician’s moral dilemma in the emergency department: going against a patient’s perceived wishes. J Emerg Med.

[39] Société Française de Médecine d’Urgence. 10ème conférence de consensus: prise en charge de la personne âgée de plus de 75 ans aux urgences. 2003. 1–19. http://www.sfmu.org/upload/consensus/pa_urgs_long.pdf. Accessed 20 Feb 2018.

[40] Tardy B, Venet C, Zeni F (2002). Death of terminally ill patients on a stretcher in the emergency department: a French speciality?. Intensive Care Med.

[41] Thuong M (2011). Recommandations sur l’information et l’abord des proches des donneurs potentiels d’organes et de tissus décédés après arrêt cardiaque (DDAC), dans l’optique d’un prélèvement. Ann Fr Med Urgence.

[42] Wall J, Hiestand B, Caterino J (2015). Epidemiology of advance directives in extended care facility patients presenting to the emergency department. West J Emerg Med.

[43] Wiese CH, Lassen CL, Bartels UE (2013). International recommendations for outpatient palliative care and prehospital palliative emergencies—a prospective questionnaire-based investigation. BMC Palliat Care.

